# You Play the Way You Train: The Influence of Accumulated Weekly Training Loads on Match Physical Performance in Soccer Players

**DOI:** 10.5114/jhk/208874

**Published:** 2026-04-02

**Authors:** Iván Asín-Izquierdo, Carlos Galiano, Fabio Y. Nakamura, Fernando H. Pareja-Blanco, Clemente Jose A. Asian-

**Affiliations:** 1 Department of ENFYRED Research Group, Musical, Plastic and Corporal Expression, Faculty of Social and Human Sciences, University of Zaragoza, Teruel, Spain.; 2 Department of Science-Based Training Research Group, Physical Performance and Sports Research Center (CIRFD), Universidad Pablo de Olavide, Seville, Spain.; 3 Department of Communication and Education, Universidad Loyola Andalucía, Seville, Spain.; 4 Research Center in Sports Sciences, Health Sciences and Human Development (CIDESD), Department of Physical Education and Sports Sciences, University of Maia, Maia, Portugal.; 5 FSI Lab, Football Science Institute, Granada, Spain.; 6 Department of Sport and Informatics, Faculty of Sport, Pablo de Olavide University, Seville, Spain.

**Keywords:** soccer, high-speed running, team sports, load monitoring, workload

## Abstract

The relationship between soccer players' match performance and cumulative training loads needs to be analysed. Therefore, the objectives of this study were: 1) to examine the influence of accumulated weekly training loads on subsequent mechanical match outcomes, and 2) to analyze individual variability in training and match loads using the coefficient of variation (CV) throughout the season. Internal and external loads over the entire season of 22 semi-professional soccer players (age 23.2 ± 4.2 years, body mass 75.0 ± 5.8 kg, and body height 1.79 ± 0.06 m) were analyzed. The devices used were 10-Hz Playertek+ GPS/GNSS units equipped with an inertial system and an accelerometer (Catapult Innovations, Melbourne, Australia) and the Borg scale in its 6–20 points version. The analysis of the data with the entire squad included all demarcations (except goalkeepers), however, in a second analysis players with the highest participation throughout the season were included, i.e., six defenders (three center back and three fullback players), three midfielders and three forwards (two wingers and one striker). Match high-speed running (HSR) showed significant correlations with most of the variables analyzed (r = 0.14–0.52). Training load variables analyzed (14.7–64.5%) exhibited larger CV values than those observed in matches (4.4–31.8%). Players with greater match HSR showed superior accumulated HSR during the week before the match than those who presented less match HSR (p = 0.024). The cumulative training load indicates a direct relationship with match HSR performance acutely and chronically. Thus, coaches should design tasks emphasizing HSR accumulation throughout the season.

## Introduction

In soccer, training and match loads are categorized as either internal or external, with the external load referring to the physical work performed, while the internal load represents the physiological and psychological stress induced by that work (Teixeira et al., 2021). Load monitoring is widely employed in soccer (Savolainen et al., 2025), with the Global Positioning System (GPS) being the most commonly used tool for monitoring the external load, as it provides metrics such as distance covered, running velocity, acceleration, and deceleration (Akenhead and Nassis, 2016). On the other hand, while physiological variables such as the heart rate, lactate concentration, and oxygen uptake have been used to assess the internal load in soccer, the rating of perceived exertion (RPE) has gained prominence due to its simplicity, accessibility, a non-invasive nature, and evidence supporting its validity and reliability (Teixeira et al., 2021).

There is no universally accepted gold standard for measuring soccer performance (Akenhead and Nassis, 2016). Therefore, it is proposed that an integrated approach to assessing internal and external loads offers more meaningful information about the stress experienced by soccer players compared to interpretations based on isolated data (Bourdon et al., 2017; Skalski et al., 2024; Szymanek-Pilarczyk et al., 2024). Numerous recent studies have used the acute:chronic workload ratio to elucidate the relationship between players’ stress and soccer performance (Bowen et al., 2017, 2020). This ratio shows the relationship between the acute load accumulated over the past seven days and the chronic workload, which is calculated as the average load over the previous four weeks, although there are other proposals with different numbers of weeks (Blanch and Gabbett, 2016). This index calculates whether the individual or team´s acute workload is greater than, lower than, or equal to the preceding chronic workload they have experienced (Bowen et al., 2017). The literature indicates a “sweet spot” between 0.8 and 1.3 that optimizes performance while minimizing the risk of injury to players (Gabbett, 2016). Despite the substantial body of literature on the acute:chronic workload ratio, the effectiveness of this method for preparing soccer players and predicting injuries remains inconsistent (Impellizzeri et al., 2020).

The lack of consistency may partly arise from the ratio's dependence on the volume of weekly training sessions and the diverse training methodologies employed by coaches (Clemente et al., 2019a). For this reason, some authors have suggested that coaches should consider the accumulated weekly load to ensure that players can effectively meet the high-intensity demands of competition (Chena et al., 2021). This approach differs from the acute:chronic workload ratio because it aims to prepare players for competition by exposing them to appropriate workloads during match preparation. Despite claims that the accumulated load during the week should be equivalent to that of a match (Malone et al., 2018), it can be two to four times greater than match values (Chena et al., 2021; Clemente et al., 2019a). Although it is acknowledged that training loads are typically higher than those experienced during competition, there is currently a lack of understanding regarding how players' performance in matches relates to the training load accumulated in preceding weeks. To the authors' knowledge, only one study has correlated the accumulated load of each week with the subsequent match demand, revealing trivial-to-small correlations between them (Clemente et al., 2019a).

Usually, the accumulated load experienced by players during training and matches is conditioned by the technical-tactical requirements (Bradley and Ade, 2018), resulting in significant variations between training weeks (Teixeira et al., 2021). The literature documents significant variability in match requirements, both between and within players, depending on the match context and the positional role (Bush et al., 2015; Carling et al., 2016). A recent study using data from the FIFA World Cup in Qatar 2022 reported that the coefficient of variation (CV) for total distance (TD), distance covered faster than 20 km·h^−1^, and distance covered faster than 25 km·h^−1^ can reach up to 12%, 35.9%, and 67.8%, respectively (Bradley, 2024).

Similarly, it has been suggested that soccer players’ responses to training vary significantly (Makar et al., 2023), as there are many possibilities for manipulating task constraints that can influence this variability (Riboli et al., 2023), highlighting the constraints of the playing space, the number of players, time, feedback, rules, and the presence or absence of goalkeepers (Asín-Izquierdo et al., 2024; de Dios-Álvarez et al., 2025; Jastrzębski et al., 2025). Despite the existing literature, conclusions remain inconclusive, with some authors confirming high variability in high-intensity locomotor demands between and within sessions throughout the season, along with small-to-moderate within-session variability in TD and distances covered at low speeds (Clemente et al., 2019a), while others report lower variability (Custódio et al., 2022). Considering this, it seems necessary to evaluate variability and changes within individual player variation to foster greater consensus in the literature on this topic (Makar et al., 2023; Trewin et al., 2018).

To the authors' knowledge, the literature presents inconsistent findings regarding the variability of the mechanical responses of soccer players during training throughout the week. In addition, analyzing how specific weekly loads may impact players' mechanical responses during matches will contribute to optimizing training load management. Therefore, the objectives of this study were: 1) to examine the influence of accumulated perceptual and mechanical weekly training loads on subsequent mechanical match outcomes, and 2) to analyze individual variability in training and match loads using the CV throughout the season.

## Methods

### Participants

The study was conducted in a Spanish semi-professional soccer team during the 2020/2021 season. The team consisted of 22 semi-professional soccer players (age 23.2 ± 4.2 years, body mass 75.0 ± 5.8 kg, and body height 1.79 ± 0.06 m) competing in the Spanish Third Division. The demarcation of the players was: two goalkeepers, eight defenders (four center backs and four full backs), six midfielders and six forwards (four wingers and two strikers). All players had at least ten years of soccer training experience and were accustomed to the tools used in the study. Soccer players could be classified within Tier 3: Highly Trained/National Level in the overall classification proposed by McKay et al. (2022). Participants were informed about the use of research data, and their involvement was voluntary and anonymous, which was confirmed by signing an informed consent form. The study was approved by the University of Maia Ethics Committee, Maia, Portugal (approval code: 210/2024; approval date: 28 May 2024) and conducted according to the Declaration of Helsinki guidelines.

### Measures

The devices used were 10-Hz Playertek+ GPS/GNSS equipped with an inertial system featuring a 400-Hz sampling rate (recorded at 100 Hz) and +/− 18-g accelerometer samples at 400 Hz in three axes (Catapult Innovations, Melbourne, Australia). The variables used to assess the internal and external load of players were the RPE and GPS-based data. The Borg scale used to measure the RPE throughout the season was the 6–20-point version (Borg, 1998), which was multiplied by the duration of the training session or the time spent participating in the match (in minutes), resulting in the session-RPE (s-RPE; au) (Arney et al., 2019). The RPE was asked individually 30 min after the training session or the match ended. For GPS variables, TD (m and km), distance covered faster than or equal to 21 km·h^−1^, understood as high-speed running (HSR; m and km), the total number of accelerations (ACC; n) and decelerations (DEC; n), and Player Load (PL; au), calculated by a mathematical formula based on the sum of data obtained by the accelerometer in all planes, were analyzed.

### Design and Procedures

A retrospective observational study was carried out to analyze correlations between internal and external training and match loads over an entire season. Data were recorded throughout all training sessions (n = 102) and matches (n = 24). Pre-season data were excluded due to their heterogeneity and limitations, focusing the analysis on the regular competitive period. The design, the procedure, and data recording operation were monitored by an external evaluator, independent of the club staff, to ensure they were carried out under optimal conditions. The usual weekly schedule consisted of four training sessions of approximately 90 minutes each, following a similar structure on Tuesday, Wednesday, Thursday, and Friday, along with one match per week (on Saturday or Sunday). Substitutes also participated in a brief post-match compensatory training session on match days. Extraordinary weeks without competition and congested weeks were included in the analyses and evaluated similarly to the rest.

Thirty minutes before the start of the warm-up, GPS-GNSS vests were assigned to the players, coinciding when they arrived at the technical room for weighing. We removed the devices and recorded the data at the end of the training session or the match, analyzing each measurement separately. The RPE was collected from the soccer players after the session or the match. GPS-GNSS data were transferred to the Go Playertek+ tool (Playertek Sync software, v 5.66; Catapult Innovations, Melbourne, Australia) and aggregated into spreadsheets for further analysis alongside the RPE data.

**Figure 1:**
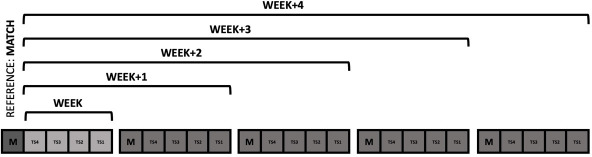
. Graphical explanation of the structure of the study by weeks. Notes: TS = Training session; M = Match

For the organization of the variables, we established five blocks of the accumulated load: the accumulated load of the week before the game (weekly) and the workload over the previous four weeks. Data were collected as follows: Weekly, Week+1, Week+2, Week+3, and Week+4, where Week+1 represented the accumulation of the current week's training load plus the previous whole week, Week+2 was the current week's training load plus the two last weeks and so on (Figure 1). Once the data for each period were obtained, all variables were correlated with the competition data. First, with the data of the entire squad (excluding, goalkeepers), a correlation analysis was conducted using all the variables related to external and internal loads during matches and the accumulated weekly sessions. Afterward, a second analysis was conducted on the players participating more in matches (n = 12) throughout the season. The inclusion criteria for these more frequently competing players were as follows: (1) they had to participate in all training sessions and matches (consecutively or not) for at least 10 weeks during the entire season, and (2) they had to play a minimum of 75 min in each match. A new analysis was carried out with this subset of higher-participation players. This included re-assessing the correlations initially done for the whole team, analyzing the variation of the studied variables across different weeks and matches, and comparing the players' weekly high-speed running (HSR) load. The comparison of the HSR load was based on whether they engaged in low, average, or high levels of HSR activity during matches (referenced as lower vs. average vs. greater).

## Statistical Analysis

All data are reported as mean values ± standard deviation (SD). The distribution of each variable was verified using the Shapiro-Wilk normality test. The absolute intra-subject variability of match and training mechanical data was assessed by the standard error of measurement, expressed in relative terms as the CV. Relationships between variables were determined using Pearson’s coefficients (r). The correlation values were qualitatively interpreted as follows: < 0.1 (trivial); ≥ 0.1 (small), ≥ 0.3 (moderate), ≥ 0.5 (large), ≥ 0.7 (very large) and ≥ 0.9 (nearly perfect) (Hopkins et al., 2009). Differences in match HSR outcomes were analyzed using a one-way ANOVA, categorizing the groups by the quantity of HSR demands (lower vs. average. vs. greater). The same analysis was applied to compare the mechanical demands of the previous week based on HSR outcomes in the following match. Threshold values for assessing magnitude of the Cohen’s *d* (*d*) effect size (ES, 90% CI) were interpreted using the following ranges: < 0.20 (trivial), ≥ 0.20 (small), ≥ 0.60 (moderate), ≥ 1.2 (large) and ≥ 2.0 (very large) (Hopkins et al., 2009). Statistical analysis was conducted using JASP software (JASP Team 2019, Version 0.18.2.0, University of Amsterdam).

## Results

### Correlation Analysis

The average values obtained for weekly training load data were as follows: s-RPE (5368.1 ± 1304.1 au), TD (21,731.2 ± 5,444.8 m), HSR (678.5 ± 383.2 m), ACC (909.9 ± 264.7), DEC (866.1 ± 254.6), and PL (1,104.2 ± 255.8 au). For match data, the average values were: TD (9,782.3 ± 1,345.3 m), HSR (492.1 ± 182.4 m), ACC (464.5 ± 83.9), DEC (447.4 ± 79.9), and PL (437.4 ± 58.2 au). Team correlation values between the mechanical variables analyzed during training and matches are described in Table 1. Match HSR showed correlations with most of the variables analyzed, displaying significant values with all variables during the current week (r = 0.22–0.40; *p* < 0.01; small to moderate). Almost all accumulated training values showed significant correlations with match HSR (r = 0.14–0.52; *p* < 0.05; small to large). Match TD showed significant but small correlations with the current week’s s-RPE (r = 0.16; *p* < 0.05) and ACC and DEC during Week+3 and Week+4 (r = 0.14–0.16; *p* < 0.05). Match ACC and DEC showed significant relationships with TD and PL during the previous week (r = −0.17–−0.15; *p* < 0.05; small), the s-RPE during Week+1 to Week+4 (r = −0.16–−0.14; *p* < 0.05; small) and PL during Week+1 (r = −0.16; *p* < 0.05; small). No significant relationships were observed for match PL.

**Table 1:** Correlation coefficients describing the relationship between match mechanical outcomes and the accumulated mechanical and perceived loads.

Week	Match-TD	Match-HSR	Match-ACC	Match-DEC	Match-PL
Week s-RPE	0.16*	0.22**	−0.01	0.00	0.04
Week+1 s-RPE	0.04	0.12	−0.13	−0.14*	−0.09
Week+2 s-RPE	0.03	0.14*	−0.15*	−0.15*	−0.09
Week+3 s-RPE	0.01	0.13	−0.16*	−0.16*	−0.11
Week+4 s-RPE	−0.01	0.14*	−0.16*	−0.16*	−0.12
Week TD	−0.01	0.31***	−0.15*	−0.16*	−0.10
Week+1 TD	<0.01	0.24***	−0.13	−0.14	−0.09
Week+2 TD	0.04	0.24***	−0.10	−0.10	−0.04
Week+3 TD	0.06	0.23***	−0.09	−0.08	−0.03
Week+4 TD	0.07	0.24***	−0.07	−0.07	−0.02
Week HSR	0.09	0.34***	0.00	−0.01	0.05
Week+1 HSR	0.10	0.52***	−0.02	−0.03	0.06
Week+2 HSR	0.12	0.50***	<0.01	<0.01	0.09
Week+3 HSR	0.12	0.48***	0.01	0.01	0.09
Week+4 HSR	0.12	0.49***	0.01	0.00	0.08
Week ACC	0.10	0.39***	0.01	0.00	0.03
Week+1 ACC	0.10	0.29***	0.04	0.03	0.03
Week+2 ACC	0.14*	0.29***	0.07	0.07	0.07
Week+3 ACC	0.16*	0.28***	0.09	0.09	0.08
Week+4 ACC	0.16*	0.28***	0.10	0.09	0.08
Week DEC	0.08	0.38***	−0.01	−0.02	0.02
Week+1 DEC	0.08	0.28***	0.03	0.02	0.02
Week+2 DEC	0.13	0.28***	0.06	0.06	0.06
Week+3 DEC	0.15*	0.27***	0.08	0.08	0.07
Week+4 DEC	0.14*	0.27***	0.09	0.09	0.07
Week PL	−0.02	0.40***	−0.17*	−0.17*	−0.07
Week+1 PL	−0.02	0.32***	−0.16*	−0.16*	−0.06
Week+2 PL	0.02	0.32***	−0.13	−0.12	−0.02
Week+3 PL	0.04	0.30***	−0.11	−0.10	0.00
Week+4 PL	0.05	0.31***	−0.09	−0.09	0.01

Notes: Week: accumulated load during the week of the match, excluding the match data; Week+1, 2, 3, and 4: chronic load from 1, 2, 3, or 4 weeks before the match, incorporating the 'week' variable. *: p < 0.05; **: p < 0.01; ***: p < 0.001. s-RPE: Session rating of perceived exertion; TD: Total distance; HSR: Distance covered at high-speed running; ACC: Accelerations; DEC: Decelerations; PL: Player load

**Figure 2:**
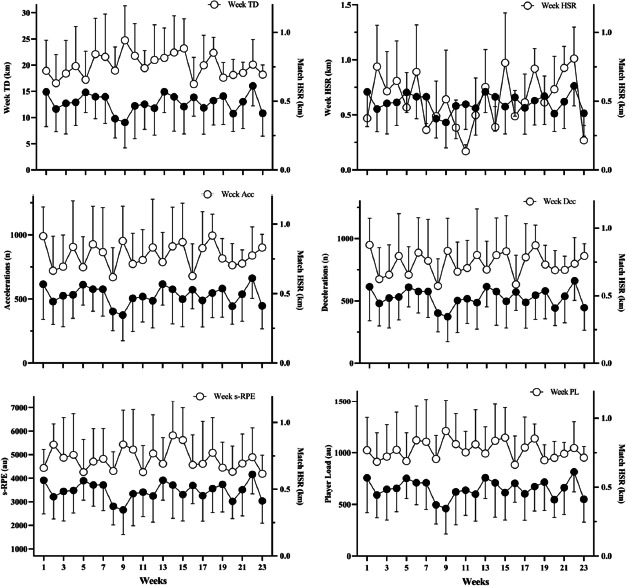
Mean ± SD descriptive representation between distance covered at high-speed running (HSR) during the matches and the weekly mechanical load for the 23 competitive weeks. Black circles represent match HSR data. Notes: s-RPE: Session rating of perceived exertion; TD: Total distance; HSR: Distance covered at high-speed running

Individual correlation values between HSR and the mechanical variables analyzed during training and matches are described in Table 2. Likewise, Figure 2 presents the descriptive values of the variables measured weekly compared to the match HSR outcomes for the corresponding week.

**Table 2:** Individual correlation coefficients describing the relationship between match mechanical outcomes and the accumulated mechanical and perceived loads.

Match HSR												
Week	Player 1	Player 2	Player 3	Player 4	Player 5	Player 6	Player 7	Player 8	Player 9	Player 10	Player 11	Player 12
Week s-RPE	0.31	0.63*	0.56*	0.17	0.23	0.22	−0.36	0.42	−0.01	0.05	0.08	0.18
Week+1 s-RPE	0.38	−0.03	0.13	0.51*	0.31	0.19	0.30	0.52	−0.39	0.01	0.46	0.23
Week+2 s-RPE	0.51	−0.11	0.03	0.28	0.28	0.44	0.58*	0.51	−0.56*	0.04	0.74**	0.36
Week+3 s-RPE	0.43	−0.12	−0.04	0.16	−0.10	0.58*	0.74**	0.37	−0.43	0.18	0.76**	0.41
Week+4 s-RPE	0.29	0.02	−0.03	0.10	−0.13	0.65*	0.72**	0.27	−0.39	0.21	0.60*	0.41
Week TD	0.41	0.15	0.61**	0.02	0.22	−0.31	−0.01	0.23	0.23	−0.03	−0.01	0.11
Week+1 TD	0.31	−0.18	0.16	0.44	0.30	−0.16	0.43	0.27	−0.33	0.07	0.35	0.01
Week+2 TD	0.51*	−0.24	0.05	0.20	0.26	0.22	0.61*	0.46	−0.50	0.20	0.65*	0.23
Week+3 TD	0.43	−0.17	−0.09	0.09	−0.01	0.42	0.73**	0.30	−0.35	0.35	0.7**	0.28
Week+4 TD	0.28	0.11	−0.13	0.13	−0.11	0.53	0.68*	0.23	−0.33	0.34	0.53	0.17
Week HSR	0.31	−0.10	−0.09	0.10	0.13	0.06	0.54	−0.15	0.30	0.22	0.12	0.32
Week+1 HSR	0.61*	−0.21	−0.20	0.39	0.40	0.53	0.58*	0.06	−0.11	0.29	0.36	0.37
Week+2 HSR	0.52*	−0.34	−0.27	0.13	0.39	0.55	0.55*	0.20	−0.33	0.26	0.59*	0.13
Week+3 HSR	0.23	−0.46	−0.35	0.19	0.15	0.63*	0.55	0.04	−0.26	0.34	0.44	0.02
Week+4 HSR	0.24	−0.39	−0.40	0.17	0.07	0.64*	0.43	0.01	−0.22	0.27	0.45	−0.08
Week ACC	0.49	0.09	0.26	−0.06	0.41	0.09	0.22	0.17	0.02	−0.04	−0.12	0.21
Week+1 ACC	0.18	−0.22	0.01	0.46	0.43	0.13	0.58*	0.17	−0.39	0.10	0.32	0.16
Week+2 ACC	0.43	−0.25	−0.02	0.17	0.35	0.42	0.65*	0.41	−0.47*	0.26	0.66*	0.35
Week+3 ACC	0.33	−0.19	−0.12	0.09	0.17	0.52	0.73**	0.28	−0.31	0.40	0.76**	0.26
Week+4 ACC	0.26	0.07	−0.15	0.17	0.04	0.61*	0.68*	0.20	−0.30	0.36	0.59*	0.15
Week DEC	0.49	0.10	0.26	−0.07	0.45	−0.05	0.21	0.21	0.07	−0.04	−0.10	0.15
Week+1 DEC	0.17	−0.20	0.02	0.45	0.46	0.06	0.57*	0.21	−0.38	0.10	0.35	0.12
Week+2 DEC	0.43	−0.24	−0.01	0.17	0.35	0.38	0.64*	0.44	−0.47*	0.26	0.68**	0.30
Week+3 DEC	0.32	−0.16	−0.11	0.10	0.16	0.48	0.73**	0.31	−0.31	0.40	0.76**	0.21
Week+4 DEC	0.26	0.10	−0.14	0.17	0.03	0.60	0.68*	0.21	−0.30	0.36	0.58*	0.11
Week PL	0.50	0.16	0.52*	0.05	0.28	−0.33	0.09	0.19	0.19	−0.01	−0.05	0.16
Week+1 PL	0.40	−0.24	0.11	0.48	0.33	−0.21	0.47	0.25	−0.34	0.08	0.34	−0.01
Week+2 PL	0.54*	−0.28	0.02	0.22	0.20	0.22	0.60*	0.44	−0.46*	0.20	0.67*	0.21
Week+3 PL	0.45	−0.17	−0.11	0.11	−0.06	0.40	0.71**	0.29	−0.33	0.34	0.71**	0.24
Week+4 PL	0.29	0.10	−0.15	0.18	−0.15	0.52	0.66*	0.22	−0.32	0.33	0.53	0.12

Notes: Week: accumulated load during the week of the match, excluding the match data; Week+1, 2, 3 and 4: chronic load from 1, 2, 3, or 4 weeks prior to the match, incorporating the 'week' variable. *: p < 0.05; **: p < 0.01; ***: p < 0.001. s-RPE: Session rating of perceived exertion; TD: Total distance; HSR: Distance covered at high-speed running; ACC: Accelerations; DEC: Decelerations; PL: Player load. Usual demarcation of soccer players: Defender (Center back: 7, 11, 12; Fullback: 2, 6, 9), Midfielder: 1, 4, 10 and Forward (Winger: 3, 8; Striker: 5)

### Variability Analysis

Training exhibited larger CV values than those seen in matches (Table 3). CV values during training ranged from 14.7% for TD to 64.5% for HSR. In contrast, matches showed CV values ranging from 4.4% for TD to 31.8% for HSR.

**Table 3:** Coefficients of variation (CV) for mechanical and perceived load variables during training and matches.

	TRAINING CV (%)												
	Player 1	Player 2	Player 3	Player 4	Player 5	Player 6	Player 7	Player 8	Player 9	Player 10	Player 11	Player 12	Avg.
s-RPE	18.3	25.9	22.0	22.9	20.3	23.9	44.3	20.5	19.3	32.3	18.9	17.0	23.8
Total Distance	24.4	27.5	26.1	29.8	24.2	21.1	26.5	14.7	21.5	27.4	25.3	25.9	24.5
HSR	51.8	56.7	47.4	51.6	44.9	58.4	54.2	50.1	47.9	64.0	64.5	61.4	54.4
Acceleration	25.6	29.8	27.7	30.1	26.9	23.8	32.0	16.5	24.8	26.0	32.6	26.0	26.8
Deceleration	26.7	30.0	29.4	31.0	27.3	24.6	32.6	17.9	26.1	25.4	32.2	24.7	27.3
Player Load	21.6	26.5	23.4	26.6	22.7	19.1	26.1	12.8	19.1	26.5	23.1	23.2	22.6
	MATCH CV (%)												
	Player 1	Player 2	Player 3	Player 4	Player 5	Player 6	Player 7	Player 8	Player 9	Player 10	Player 11	Player 12	Avg.
Total Distance	9.7	14.7	8.2	11.3	6.0	13.5	6.8	18.4	13.3	13.9	4.4	4.7	10.4
HSR	15.5	28.3	17.4	18.3	20.1	29.8	31.8	22.8	19.2	23.1	29.9	27.9	23.7
Acceleration	9.2	12.0	10.3	11.7	6.6	12.0	8.4	20.8	12.6	11.2	5.8	7.6	10.7
Deceleration	9.3	12.0	11.2	12.0	6.9	11.0	7.6	22.6	12.6	12.5	5.2	6.4	10.8
Player Load	8.7	12.6	7.0	10.1	4.7	13.0	6.5	16.3	10.6	13.1	5.9	4.9	9.5

Notes: s-RPE: Session rating of perceived exertion; TD: Total distance; HSR: Distance covered at high-speed running; ACC: Accelerations; DEC: Decelerations; PL: Player load. Usual demarcation of soccer players: Defender (Center back: 7, 11, 12; Fullback: 2, 6, 9), Midfielder: 1, 4, 10 and Forward (Winger: 3, 8; Striker: 5)

### Match HSR Response Analysis

**Figure 3:**
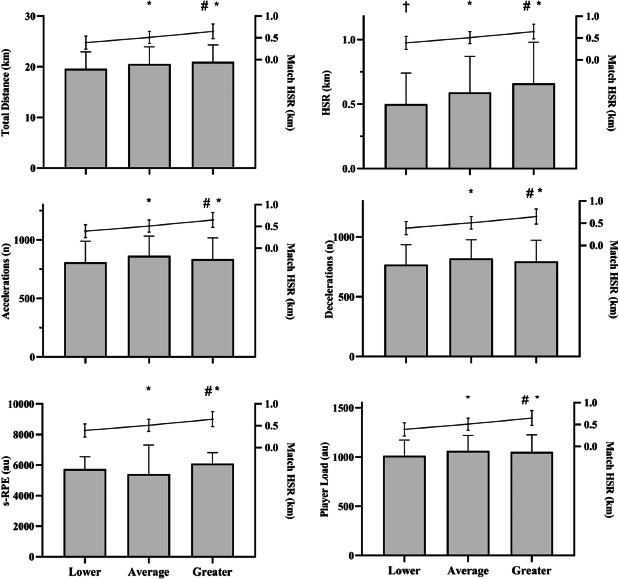
. Comparison between the week load during the season when distance covered at high-speed running (HSR) during the matches was greater, lower or average. Notes: *: p < 0.05 for HSR data vs. low; #: p < 0.05 for HSR data vs. average; †: p < 0.05 for week HSR vs. greater. Lines represent match HSR. Bars represent the variable described in the left y axis. s-RPE: Session rating of perceived exertion; HSR: Distance covered at high-speed running

Differences in the mechanical response from the previous week based on match HSR outcomes, depicting the greatest, lowest, and average values, are shown in Figure 3. Significant differences were observed when match HSR was low compared to both average and high HSR outcomes (ES > 0.79; *p* < 0.001). Furthermore, significant differences were observed when match HSR was high compared to both average and low HSR outcomes (ES > 0.87; *p* < 0.001). In weeks when HSR during matches was high, weekly HSR was significantly greater (ES = 0.58; *p* = 0.024) compared to weeks with low HSR during matches.

## Discussion

The main objectives of this study were 1) to examine the influence of the accumulated perceptual and mechanical training loads on match outcomes, and 2) to analyze individual variability of training and match loads throughout the season. After analyzing mechanical and perceptual data from the entire season, the study's findings were as follows: (i) HSR during matches significantly correlated with accumulated perceptual and mechanical loads; however, these correlations were no longer significant when analyzing individual data from players who participated in a greater number of matches; (ii) training loads exhibited greater variability compared to matches; (iii) when grouping weeks based on the HSR distance covered during matches, significant differences in the weekly HSR load were observed.

Understanding the cumulative impact of training loads on match mechanical performance is crucial for optimizing each player's performance. Our study revealed significant correlations between the accumulated training load and match mechanical response (Table 1). Specifically, the accumulated HSR load showed significant relationships with match mechanical response. Furthermore, the accumulated HSR load showed the highest correlation values with match HSR, indicating a positive relationship. For instance, HSR data from Week+1 showed a significant correlation with match HSR (r = 0.52), suggesting that players who accumulated greater HSR distance in the preceding and current weeks tended to cover more distance at HSR during the match. A recent study also demonstrated a significant correlation between match mechanical performance and the accumulated load from the same week (Clemente et al., 2019a). We could assume that as the season progressed and players’ performance improved, the weekly workload increased, which should be reflected in superior mechanical output during matches. However, this statement can be challenged, as there was no observable progression in workloads within the team, nor were there temporal increases in mechanical responses during matches (Figure 2). Previous research also indicated increases in mechanical response during matches when the accumulated load exceeded the team average (Lazarus et al., 2017). This suggests the need to adjust players’ training loads based on match participation and to ensure sufficient exposure to the sprint and HSR loads for players with limited match exposure, for example, through the use of transition games specifically designed to target these variables (Asian-Clemente et al., 2022, 2023, 2024). Other authors identified a linear relationship between a chronic high metabolic load and a high metabolic load in matches, suggesting that high chronic exposure to high-intensity activity led to an increase in high-intensity actions during matches (Springham et al., 2020). It has also been indicated that elevated chronic workloads enhance readiness among professional soccer players and reduce injury risk (Bowen et al., 2020). Our study shows that the high accumulated load in previous weeks may indicate match HSR performance in the upcoming matches, given the positive and large correlation observed.

However, the results shifted when we performed the individual correlation analysis for players who played at least 10 games during the season (Table 2). Statistical significance was limited, with only players 7 and 11 demonstrating a significant correlation between accumulated and match mechanical loads. The high heterogeneity in mechanical variables across matches, particularly when involving the entire squad, contributes to significant correlations. These results highlight the necessity of individualizing training loads throughout the season to improve the stimulus-response continuum. As indicated by the findings, some players demonstrated a greater capacity to perform when exposed to chronic higher training loads, while others exhibited the opposite response. Given that only 12 players consistently participated in a higher number of matches, this limitation underscores the need for future research to explore the accumulated load and match responses with larger sample sizes. Nevertheless, significant differences were observed between matches with higher, lower, and average HSR when analyzing players with more frequent game participation. Specifically, when players covered greater distances in HSR during the match, significant differences were noted in the previous week's accumulated HSR compared to weeks with lower match HSR (Figure 3). However, it should be noted that excessive acute loads might negatively impact the player performance (Soligard et al., 2016), potentially leading to adverse effects on their physical development (Douchet et al., 2024). Therefore, the average and slightly above-average values in the acute accumulated load appear to be associated with a higher performance response in competition. However, this must be monitored in relation to physical performance, considering both fitness and fatigue levels (Gabbett, 2016). Indeed, a new question arises from this finding. Did they present greater HSR during matches due to the HSR accumulated in the previous weeks? Or did they present higher HSR during matches and training sessions because they were fitter? Further studies should examine these research questions.

Understanding variability in training and match loads can assist coaches in programming players’ stimuli throughout the season (Martín-García et al., 2018; Varjan et al., 2024) and identify changes in performance that are typical, as well as those that deviate from the norm (Oliva-Lozano et al., 2021). This investigation revealed great differences in CV values between training sessions and match play for all measured variables (Table 3). The heightened CV observed during training sessions may be attributed to the dynamic nature of microcycle load management, which fluctuates significantly based on proximity to match days and the target of the training tasks (Martín-García et al., 2018). For instance, a study comparing daily variability within the week and the match among professional players (Oliva-Lozano et al., 2023) demonstrated that each training day exhibited distinct variability patterns. Notably, the day preceding a match showed the lowest variability, significantly lower than that observed on the match day itself.

When comparing the remaining variables of this study with previously published results, the findings exhibit diversity. While training sessions showed better reproducibility for TD, accelerations, decelerations and PL, there was significant variation in other external load components, particularly in HSR and sprinting. CV values for these variables vary widely across studies, with HSR showing variations ranging from approximately 20% to over 60% (Clemente et al., 2019b; Dello Iacono et al., 2023; Oliva-Lozano et al., 2021). The mechanical load, encompassing accelerations and decelerations, exhibited a CV of approximately ≈27% in our study, which is higher than variability reported in most studies where those values typically ranged from about 8% to 12% (Aquino et al., 2019; Stevens et al., 2016). However, these results are consistent with previous research (Younesi et al., 2021) which reported that mechanical load variability could reach up to 22.5%, depending on the type of the task. Similarly, PL also demonstrated higher CV values in our study compared to previous findings in a study conducted using small sided-games, where the CV ranged from 4.9 to 6.0% (Clemente et al., 2019b). These discrepancies between our findings and previously published literature may be attributed to the fact that we examined complete micro-cycles, while other studies focused on variability within specific training tasks designed with particular constraints. Considering that task constraints and characteristics appear to influence players' physical stimuli (Dello Iacono et al., 2023), this could have been a decisive factor in yielding different results. This heterogeneity in the players' external load could explain the high variability of the s-RPE found in our study (23.8%). Although a previous review on small sided-games reported higher CVs for players' perceived exertion responses (CV = 36.2–128.4%), the authors acknowledged the limited evidence on this variable in the literature, highlighting the need for further scientific investigations (Clemente et al., 2022).

Regarding the variability observed in match data, the findings of this study align with previous literature, demonstrating that TD, accelerations and decelerations exhibited a low-moderate CV (Rabbani et al., 2024; Thoseby et al., 2022; Trewin et al., 2018). Additionally, as the velocity of movement increased, the CV also tended to rise. The heterogeneity among studies analyzing variability in matches complicates inter-study comparisons, with our data yielding diverse results depending on the selected variables. For instance, with regard to TD, we found variability of ≈10%, which is consistent with findings from previous research involving professional players (Rabbani et al., 2024). Regarding HSR, all studies reported the highest CVs for this variable, with our values aligning closely with those found by previous authors (Thoseby et al., 2022). The variability observed in accelerations and decelerations also presents a diverse profile. In our study, both variables exhibited a CV of ≈10%, whereas previous studies reported variability ranges for these measures between 5% and 21% (Rabbani et al., 2024; Thoseby et al., 2022; Trewin et al., 2018). Lastly, to the authors' knowledge, only one article has analyzed the variability of PL in matches (Trewin et al., 2018), which reported higher values than those found in the present study (14% vs. 9.5%).

This study has several limitations, primarily from its implementation within a semi-professional soccer team setting. Notable constraints include the complex interaction of variables and the small sample size. Data selection choices, such as excluding certain match participations or players who did not meet specific criteria, might impact the results. Sole reliance on the s-RPE for analyzing the internal load and overlooking contextual variables such as the player position, the match location, and the opponent’s performance level are further limitations. Further research should be carried out in the future with soccer players of different age and performance levels.

In conclusion, our results demonstrate significant correlations between match HSR distance and the accumulated training load. However, intra-individual correlation analyses involving players with more frequent participation yielded less consistent results. Nevertheless, when these players covered greater HSR distances during matches, their HSR values from the previous week were significantly higher compared to matches where they engaged in low HSR. These results are conditioned by the considerable variability in the weekly load and match performance throughout the season, being higher during the training week/micro-cycle compared to the matches. These findings contribute to a deeper understanding of the relationship between training loads and match performance, providing valuable insights into how training can be optimized for better outcomes.

### Practical Implications

This study offers coaches, researchers, and soccer professionals valuable practical insights. The findings align with previous research, demonstrating a positive correlation between training loads and match performance, especially regarding HSR. Maintaining consistent HSR and sprinting loads throughout the season may enhance competitive performance. Utilizing load indices in relation to match performance is recommended, as weekly accumulated loads are typically higher and exhibit greater variability than match loads. The significant variability in both matches and training, coupled with its highly individual nature, highlights the need for tailored approaches. Overall, the cumulative training load of the team has a direct relationship with match HSR performance, both acutely and chronically, within a complex context influenced by numerous factors throughout the season.

## References

[ref1] Akenhead, R., & Nassis, G. P. (2016). Training Load and Player Monitoring in High-Level Football: Current Practice and Perceptions. *International Journal of Sports Physiology and Performance*, 11(5), 587–593. 10.1123/ijspp.2015-0331 26456711

[ref2] Aquino, R., Melli-Neto, B., Ferrari, J. V. S., Bedo, B. L. S., Vieira, L. H. P., Santiago, P. R. P., Gonçalves, L. G. C., Oliveira, L. P., & Puggina, E. F. (2019). Validity and reliability of a 6-a-side small-sided game as an indicator of match-related physical performance in elite youth Brazilian soccer players. *Journal of Sports Sciences*, 37(23), 2639–2644. 10.1080/02640414.2019.1608895 31064264

[ref3] Arney, B. E., Glover, R., Fusco, A., Cortis, C., de Koning, J. J., van Erp, T., Jaime, S., Mikat, R. P., Porcari, J. P., & Foster, C. (2019). Comparison of RPE (Rating of Perceived Exertion) Scales for Session RPE. *International Journal of Sports Physiology and Performance*, 14(7), 994–996. 10.1123/ijspp.2018-0637 30569764

[ref4] Asian-Clemente, J. A., Rabano-Muñoz, A., Requena, B., & Suarez-Arrones, L. (2022). High-speed Training in a Specific Context in Soccer: Transition Games. *International Journal of Sports Medicine*, 43(10), 881–888. 10.1055/a-1794-9567 35272387

[ref5] Asian-Clemente, J. A., Rabano-Muñoz, A., Suarez-Arrones, L., & Requena, B. (2023). Different pitch configurations constrain the external and internal loads of young professional soccer players during transition games. *Biology of Sport*, 40(4), 1047–1055. 10.5114/biolsport.2023.124848 37867736 PMC10588570

[ref6] Asian-Clemente, J., Rabano-Muñoz, A., Suarez-Arrones, L., & Requena, B. (2024). Analysis of Differences in Running Demands between Official Matches and Transition Games of Young Professional Soccer Players according to the Playing Position. *Journal of Human Kinetics*, 92(1), 121–131. 10.5114/jhk/175339 38736606 PMC11079932

[ref7] Asín-Izquierdo, I., Gutiérrez-García, L., & Galiano, C. (2024). Application of technology for the analysis of Small-Sided Games in football. From complexity to chaos in training design: Reference to number of players, playing space, orientation, time distribution, directionality with goalkeepers, and feedback. *Proceedings of the Institution of Mechanical Engineers, Part P: Journal of Sports Engineering and Technology*, 238(2), 117–125. 10.1177/17543371231175946

[ref8] Blanch, P., & Gabbett, T. J. (2016). Has the athlete trained enough to return to play safely? The acute:chronic workload ratio permits clinicians to quantify a player’s risk of subsequent injury. *British Journal of Sports Medicine*, 50(8), 471–475. 10.1136/bjsports-2015-095445 26701923

[ref9] Borg G. (1998). Borg’s Perceived Exertion and Pain Scales. Human Kinetics.

[ref10] Bourdon, P. C., Cardinale, M., Murray, A., Gastin, P., Kellmann, M., Varley, M. C., Gabbett, T. J., Coutts, A. J., Burgess, D. J., Gregson, W., & Cable, N. T. (2017). Monitoring Athlete Training Loads: Consensus Statement. *International Journal of Sports Physiology and Performance*, 12(s2), 161–170. 10.1123/IJSPP.2017-0208 28463642

[ref11] Bowen, L., Gross, A. S., Gimpel, M., Bruce-Low, S., & Li, F.-X. (2020). Spikes in acute:chronic workload ratio (ACWR) associated with a 5–7 times greater injury rate in English Premier League football players: a comprehensive 3-year study. *British Journal of Sports Medicine*, 54(12), 731–738. 10.1136/bjsports-2018-099422 30792258 PMC7285788

[ref12] Bowen, L., Gross, A. S., Gimpel, M., & Li, F.-X. (2017). Accumulated workloads and the acute:chronic workload ratio relate to injury risk in elite youth football players. *British Journal of Sports Medicine*, 51(5), 452–459. 10.1136/bjsports-2015-095820 27450360 PMC5460663

[ref13] Bradley, P. S. (2024). ‘Setting the Benchmark’ Part 1: The Contextualised Physical Demands of Positional Roles in the FIFA World Cup Qatar 2022. *Biology of Sport*, 41(1), 261–270. 10.5114/biolsport.2024.131090 38188125 PMC10765430

[ref14] Bradley, P. S., & Ade, J. D. (2018). Are Current Physical Match Performance Metrics in Elite Soccer Fit for Purpose or Is the Adoption of an Integrated Approach Needed? *International Journal of Sports Physiology and Performance*, 13(5), 656–664. 10.1123/ijspp.2017-0433 29345547

[ref15] Bush, M. D., Archer, D. T., Hogg, R., & Bradley, P. S. (2015). Factors Influencing Physical and Technical Variability in the English Premier League. *International Journal of Sports Physiology and Performance*, 10(7), 865–872. 10.1123/ijspp.2014-0484 25671294

[ref16] Carling, C., Bradley, P., McCall, A., & Dupont, G. (2016). Match-to-match variability in high-speed running activity in a professional soccer team. *Journal of Sports Sciences*, 34(24), 2215–2223. 10.1080/02640414.2016.1176228 27144879

[ref17] Chena, M., Morcillo, J. A., Rodríguez-Hernández, M. L., Zapardiel, J. C., Owen, A., & Lozano, D. (2021). The Effect of Weekly Training Load across a Competitive Microcycle on Contextual Variables in Professional Soccer. *International Journal of Environmental Research and Public Health*, 18(10), 5091. 10.3390/ijerph18105091 34064978 PMC8151593

[ref18] Clemente, F., Aquino, R., Praça, G. M., Rico-González, M., Oliveira, R., Filipa Silva, A., Sarmento, H., & Afonso, J. (2022). Variability of internal and external loads and technical/tactical outcomes during small-sided soccer games: a systematic review. *Biology of Sport*, 39(3), 647–672. 10.5114/biolsport.2022.107016 35959343 PMC9331334

[ref19] Clemente, F. M., Rabbani, A., Conte, D., Castillo, D., Afonso, J., Truman Clark, C. C., Nikolaidis, P. T., Rosemann, T., & Knechtle, B. (2019a). Training/Match External Load Ratios in Professional Soccer Players: A Full-Season Study. *International Journal of Environmental Research and Public Health*, 16(17), 3057. 10.3390/ijerph16173057 31443592 PMC6747517

[ref20] Clemente, F. M., Rabbani, A., Kargarfard, M., Nikolaidis, P. T., Rosemann, T., & Knechtle, B. (2019b). Session-To-Session Variations of External Load Measures of Youth Soccer Players in Medium-Sided Games. *International Journal of Environmental Research and Public Health*, 16(19), 3612. 10.3390/ijerph16193612 31561570 PMC6801539

[ref21] Custódio, I. J. de O., Praça, G. M., Paula, L. V. de, Bredt, S. da G. T., Nakamura, F. Y., & Chagas, M. H. (2022). Intersession reliability of GPS-based and accelerometer-based physical variables in small-sided games with and without the offside rule. *Proceedings of the Institution of Mechanical Engineers, Part P: Journal of Sports Engineering and Technology*, 236(2), 134–142. 10.1177/1754337120987646

[ref22] de Dios-Álvarez, V., Padrón-Cabo, A., Alkain, P., Rey, E., & Castellano, J. (2025). Area per Player in Small-Sided Games to Estimate the External Load in Elite Youth Soccer Players. *Journal of Human Kinetics*, 95(1), 123–138. 10.5114/jhk/189421 39944978 PMC11812169

[ref23] Dello Iacono, A., McLaren, S. J., Macpherson, T. W., Beato, M., Weston, M., Unnithan, V. B., & Shushan, T. (2023). Quantifying Exposure and Intra-Individual Reliability of High-Speed and Sprint Running During Sided-Games Training in Soccer Players: A Systematic Review and Meta-analysis. *Sports Medicine*, 53(2), 371–413. 10.1007/s40279-022-01773-1 36331702 PMC9877094

[ref24] Douchet, T., Paizis, C., Carling, C., Babault, N. (2024). Influence of a Modified versus a Typical Microcycle Periodization on the Weekly External Loads and Match Day Readiness in Elite Academy Soccer Players. *Journal of Human Kinetics*, 93(1), 133–144. 10.5114/jhk/182984 39132417 PMC11307180

[ref25] Gabbett, T. J. (2016). The training—injury prevention paradox: should athletes be training smarter *and* harder? *British Journal of Sports Medicine*, 50(5), 273–280. 10.1136/bjsports-2015-095788 26758673 PMC4789704

[ref26] Hopkins, W., Marshall, S., Batterham, A., & Hanin, J. (2009). Progressive Statistics for Studies in Sports Medicine and Exercise Science. *Medicine & Science in Sports & Exercise*, 41(1), 3–12. 10.1249/MSS.0b013e31818cb278 19092709

[ref27] Impellizzeri, F. M., Menaspà, P., Coutts, A. J., Kalkhoven, J., & Menaspà, M. J. (2020). Training Load and Its Role in Injury Prevention, Part I: Back to the Future. *Journal of Athletic Training*, 55(9), 885–892. 10.4085/1062-6050-500-19 32991701 PMC7534945

[ref28] Jastrzębski, Z., Wakuluk-Lewandowska, D., Arslan, E., Kilit, B., Soylu, Y., & Radzimiński, Ł. (2025). Effects of Eight-Week Game-Based High-Intensity Interval Training Performed on Different Pitch Dimensions on the Level of Physical Capacity and Time-Motion Responses in Youth Soccer Players. *Journal of Human Kinetics*, 97(1), 157–168. 10.5114/jhk/190842 40463325 PMC12127915

[ref29] Lazarus, B. H., Stewart, A. M., White, K. M., Rowell, A. E., Esmaeili, A., Hopkins, W. G., & Aughey, R. J. (2017). Proposal of a Global Training Load Measure Predicting Match Performance in an Elite Team Sport. *Frontiers in Physiology*, 8(1), 930. 10.3389/fphys.2017.00930 29209229 PMC5702311

[ref30] Makar, P., Silva, A., Kawczyński, A., Akyildiz, Z., Yildiz, M., Praça, G., & Clemente, F. (2023). Variability of peak speed and sprinting actions during the same small-sided games: within-and between-player variations inspected over four consecutive weeks. *Biology of Sport*, 40(4), 959–965. 10.5114/biolsport.2023.124846 37867758 PMC10588576

[ref31] Malone, S., Owen, A., Mendes, B., Hughes, B., Collins, K., & Gabbett, T. J. (2018). High-speed running and sprinting as an injury risk factor in soccer: Can well-developed physical qualities reduce the risk? *Journal of Science and Medicine in Sport*, 21(3), 257–262. 10.1016/j.jsams.2017.05.016 28595870

[ref32] Martín-García, A., Gómez Díaz, A., Bradley, P. S., Morera, F., & Casamichana, D. (2018). Quantification of a Professional Football Team’s External Load Using a Microcycle Structure. *Journal of Strength and Conditioning Research*, 32(12), 3511–3518. 10.1519/JSC.0000000000002816 30199452

[ref33] McKay, A. K. A., Stellingwerff, T., Smith, E. S., Martin, D. T., Mujika, I., Goosey-Tolfrey, V. L., Sheppard, J., & Burke, L. M. (2022). Defining Training and Performance Caliber: A Participant Classification Framework. *International Journal of Sports Physiology and Performance*, 17(2), 317–331. 10.1123/ijspp.2021-0451 34965513

[ref34] Oliva-Lozano, J. M., Barbier, X., Fortes, V., & Muyor, J. M. (2023). Key load indicators and load variability in professional soccer players: a full season study. *Research in Sports Medicine*, 31(3), 201–213. 10.1080/15438627.2021.1954517 34259100

[ref35] Oliva-Lozano, J. M., Muyor, J. M., Fortes, V., & McLaren, S. J. (2021). Decomposing the variability of match physical performance in professional soccer: Implications for monitoring individuals. *European Journal of Sport Science*, 21(11), 1588–1596. 10.1080/17461391.2020.1842513 33100192

[ref36] Rabbani, A., Ermidis, G., Clemente, F. M., & Twist, C. (2024). Variability of External Load Measures During Soccer Match Play: Influence of Player Fitness or Pacing? *International Journal of Sports Physiology and Performance*, 19(4), 340–346. 10.1123/ijspp.2023-0243 38198797

[ref37] Riboli, A., Esposito, F., & Coratella, G. (2023). Technical and locomotor demands in elite soccer: manipulating area per player during small-sided games to replicate official match demands. *Biology of Sport*, 40(3), 639–647. 10.5114/biolsport.2023.118338 37398955 PMC10286612

[ref38] Savolainen, E. H. J., Ihalainen, J. K., & Walker, S. (2025). Female Soccer Players’ In-Season Weekly Training Load and Intensity: Comparison between National League’s Top and Bottom-Half Ranked Teams. *Journal of Human Kinetics*, 95(1), 187–198. 10.5114/jhk/189657 39944977 PMC11812167

[ref39] Soligard, T., Schwellnus, M., Alonso, J.-M., Bahr, R., Clarsen, B., Dijkstra, H. P., Gabbett, T., Gleeson, M., Hägglund, M., Hutchinson, M. R., Janse van Rensburg, C., Khan, K. M., Meeusen, R., Orchard, J. W., Pluim, B. M., Raftery, M., Budgett, R., & Engebretsen, L. (2016). How much is too much? (Part 1) International Olympic Committee consensus statement on load in sport and risk of injury. *British Journal of Sports Medicine*, 50(17), 1030–1041. 10.1136/bjsports-2016-096581 27535989

[ref40] Skalski, D., Prończuk, M., Łosińska, K., Spieszny, M., Kostrzewa, M., Aschenbrenner, P., & Maszczyk, A. (2024). The impact of asymmetry in lower limb muscle strength and power on straight-line running speed in female soccer players. *Baltic Journal of Health and Physical Activity*, 16(4), Article 6. 10.29359/BJHPA.16.4.06

[ref41] Springham, M., Williams, S., Waldron, M., Strudwick, A. J., Mclellan, C., & Newton, R. U. (2020). Prior workload has moderate effects on high-intensity match performance in elite-level professional football players when controlling for situational and contextual variables. *Journal of Sports Sciences*, 38(20), 2279–2290. 10.1080/02640414.2020.1778355 32543282

[ref42] Stevens, T. G. A., De Ruiter, C. J., Beek, P. J., & Savelsbergh, G. J. P. (2016). Validity and reliability of 6-a-side small-sided game locomotor performance in assessing physical fitness in football players. *Journal of Sports Sciences*, 34(6), 527–534. 10.1080/02640414.2015.1116709 26630259

[ref43] Szymanek-Pilarczyk, M., Nowak, M. J., Góra, T., Oleksy, Ł., Drozd, M., & Wąsik, J. (2024). The Evaluation of the Modified Wave Periodization Model Efficiency on the Example of Young Soccer Players’ Sprint Tests. *Journal of Human Kinetics*, 94(1), 215–226. 10.5114/jhk/191699 39563758 PMC11571460

[ref44] Teixeira, J. E., Forte, P., Ferraz, R., Leal, M., Ribeiro, J., Silva, A. J., Barbosa, T. M., & Monteiro, A. M. (2021). Monitoring Accumulated Training and Match Load in Football: A Systematic Review. *International Journal of Environmental Research and Public Health*, 18(8), 3906. 10.3390/ijerph18083906 33917802 PMC8068156

[ref45] Thoseby, B., D. Govus, A., C. Clarke, A., J. Middleton, K., & J. Dascombe, B. (2022). Between-match variation of peak match running intensities in elite football. *Biology of Sport*, 39(4), 833–838. 10.5114/biolsport.2022.109456 36247963 PMC9536389

[ref46] Trewin, J., Meylan, C., Varley, M. C., & Cronin, J. (2018). The match-to-match variation of match-running in elite female soccer. *Journal of Science and Medicine in Sport*, 21(2), 196–201. 10.1016/j.jsams.2017.05.009 28595867

[ref47] Varjan, M., Hank, M., Kalata, M., Chmura, P., Mala, L., & Zahalka, F. (2024). Weekly Training Load Differences between Starting and Non-Starting Soccer Players. *Journal of Human Kinetics*, 90(1), 125–131. 10.5114/jhk/171449 38380307 PMC10875698

[ref48] Younesi, S., Rabbani, A., Clemente, F., Sarmento, H., & J. Figueiredo, A. (2021). Session-to-session variations in external load measures during small-sided games in professional soccer players. *Biology of Sport*, 38(2), 185–193. 10.5114/biolsport.2020.98449 34079163 PMC8139343

